# Whole-genome selective scans detect genes associated with important phenotypic traits in goat (*Capra hircu*s)

**DOI:** 10.3389/fgene.2023.1173017

**Published:** 2023-04-18

**Authors:** Xing Wan, Jia-Nan Jing, Dong-Feng Wang, Feng-Hua Lv

**Affiliations:** ^1^ College of Animal Science and Technology, China Agricultural University, Beijing, China; ^2^ CAS Key Laboratory of Animal Ecology and Conservation Biology, Institute of Zoology, Chinese Academy of Sciences (CAS), Beijing, China; ^3^ University of Chinese Academy of Sciences (UCAS), Beijing, China

**Keywords:** goat, artificial selection, phenotypic traits, whole-genome scan, candidate genes

## Abstract

Goats with diverse economic phenotypic traits play an important role in animal husbandry. However, the genetic mechanisms underlying complex phenotypic traits are unclear in goats. Genomic studies of variations provided a lens to identify functional genes. In this study, we focused on the worldwide goat breeds with outstanding traits and used whole-genome resequencing data in 361 samples from 68 breeds to detect genomic selection sweep regions. We identified 210–531 genomic regions with six phenotypic traits, respectively. Further gene annotation analysis revealed 332, 203, 164, 300, 205, and 145 candidate genes corresponding with dairy, wool, high prolificacy, poll, big ear, and white coat color traits. Some of these genes have been reported previously (e.g., *KIT*, *KITLG*, *NBEA*, *RELL1*, *AHCY,* and *EDNRA*), while we also discovered novel genes, such as *STIM1*, *NRXN1*, *LEP*, that may be associated with agronomic traits like poll and big ear morphology. Our study found a set of new genetic markers for genetic improvement in goats and provided novel insights into the genetic mechanisms of complex traits.

## Introduction

As one of the earliest domesticated animals, goats profoundly affect human society (Porter, 1996; Pringle, 1998). Then, human beings reshaped the morphology, physiology, and behavior of goats by domestication, selection, and dispersal with humans (Larson and Fuller, 2014). Goats have formed a wealth of breed resources adapted to different natural environments and human needs in the past 10,000 years (Pereira and Amorim, 2010; Zheng et al., 2020), exhibited specialized phenotypes (e.g., coat color, horn, ear), and provided diverse productions (e.g., milk, fiber) (Skapetas and Bampidis, 2016). For the animals, those traits selected in a specific direction may be imprinted obvious characteristics in the genome (Grossman et al., 2010; Li et al., 2020; Mariadassou et al., 2020; LI et al., 2022; Seo et al., 2022).

Recent studies by integrating whole genome datasets and ancient DNA information have identified candidate genome regions and genes during goat domestication (Alberto et al., 2018; Daly et al., 2018; Zheng et al., 2020). The availability of whole-genome datasets provides a lens to uncover the genetic mechanism underlying phenotypic traits. Many candidate genes or selective regions were identified in recent studies by selective sweep or whole-genome association analysis (GWAS) using re-sequencing datasets and whole-genome SNPs arrays (Guo et al., 2018; Islam et al., 2020; Luigi-Sierra et al., 2020; Talouarn et al., 2020; Wang K. et al., 2020; Gu et al., 2022). Nevertheless, most of those studies focused on given breeds or included breeds restricted in geographic regions, and little is known regarding the genetic mechanisms of diverse phenotypic traits of goats on a worldwide scale. Here, we collected samples on the global scale with various phenotypes and aimed to conduct a genome-wide analysis to identify genomic regions associated with the phenotypic traits underlying recently strong selection. The identification of genes associated with varied agronomic traits across worldwide goats facilitates prospective molecular breeding endeavors.

## Materials and methods

### Genotypic and phenotypic data

We collected 361 samples including 68 domestic goat breeds with typical phenotypic traits (Supplementary Table S1). Whole-genome resequencing datasets of 361 samples were retrieved from the National Center for Biotechnology Information (NCBI) (Supplementary Table S1). The raw reads were filtered with Trimmomatic v0.39 (Bolger et al., 2014), and filtered reads were aligned to the goat reference genome (ARS1) by the Burrows-Wheeler Aligner v0.7.17 (Li and Durbin, 2009) with default parameters. Then we carried out the GATK Best Practices Workflows to call short variations. We filtered duplicates by the *MarkDuplicates* module with Picard v2.18.12 (http://broadinstitute.github.io/picard/) and detected short variations using the GATK v4.2.3.0 *HaplotypeCaller* module (McKenna et al., 2010) in individual level. The raw GVCF files of each sample were merged using the *CombineGVCFs* and detected for short variations using the *GenotypeGVCFs*. In this study, we only selected SNP by the *SelectVariants* module in GATK. The raw SNPs were firstly filtered by *VariantFiltering* module of the GATK with the parameters “QUAL <30.0 || QD < 2.0 || MQ < 40.0 || FS > 60.0 || SOR >3.0 || MQRankSum < −12.5 || ReadPosRankSum < −8.0”. We further identified high quality SNPs using the following criteria: (i) biallelic SNPs, (ii) autosome SNPs, (iii) minor allele frequency (MAF) > 0.05, (iv) call rate >90%. The above analyses were performed by VCFtools v0.1.14 (Danecek et al., 2011).

The total 361 samples were classified into six pairs of populations according to six important economic traits (Table 1). 88 dairy type individuals, 19 non-dairy type individuals, 24 wool type individuals, 22 wild type individuals, 14 high prolificacy individuals, 25 low prolificacy individuals, 31 poll individuals, 15 horn individuals, 14 big ear individuals, 22 small ear individuals, 45 white coat individuals, and 37 black coat individuals were obtained (Table 1).

**TABLE 1:** Information from worldwide goat breeds used to detect selective signals associated with specific traits.

Traits	Category	Populations (number of samples)	Comparisons
Milk	Dairy type	Kamori (1), Dera Din Panah (1), Maure (3), Galla (3), Sofia (7), Toggenburg (22), Saanen (20), Provencale (1), Fosses (1), Poitevine (4), Savoie (4), Alpine (8), Appenzeller (Appenzell) (13)	Dairy type *versus* Non-dairy type
	Non-dairy type	Black Bengal Goat (2), Grisons Striped (Grison Striped) (15), Rove (2)	
Wool	Wool type	Pak-Angora (Angora) (7), Liaoning cashmere goat (5)., cashmere goat (11), White Chanthangi Pashmina/Cashmere goat (1)	Wool type *versus* Wild type
	Wild type	Toggenburg (22)	
Reproduction	High prolificacy	Black Bengal Goat (2), Jining Gray goat (Qin goat) (1), Barbari (1), Naine (3), Woyito_Guji (7)	High prolificacy *versus* Low prolificacy
	Low prolificacy	Sonjo (3), Toggenburg (22)	
Horn	Poll	Matou (1), Toggenburg (22), Alpine (8)	Poll *versus* Horn
	Horn	Grisons Striped (Grison Striped) (15)	
Ear	Big ear	Pak-Angora (Angora) (7), Kamori (1), Daer (Jianyang big ear goat) (1), Matebele (5)	Big ear *versus* Small ear
	Small ear	Toggenburg (22)	
Coat Color	White	Liaoning cashmere goat (5), Maasai (2), White Chanthangi Pashmina/Cashmere goat (1), Gumez (4), cashmere goat (11), Saanen (20), Laoshan dairy goat (2)	White *versus* Black
	Black	Daer (Jianyang big ear goat) (1), Valais Blacknecked (29), Leizhou goat (5), Black Bengal Goat (2)	

### Genomic selection signals analysis

To identify the genomic signatures of selection in domestic goats with special phenotypes, we carried out two approaches. We calculated the pairwise *F*_ST_ values (Weir and Cockerham, 1984) between pairwise populations with contrasting phenotypes (Table 1). Further, we calculated ln (*θ*_π_ ratios) (*θ*π-Control/*θ*π-Case) of pairwise populations with contrasting phenotypes to detect changes in genetic diversity due to artificial selection (Yang et al., 2016). The above analyses were all performed by VCFtools v0.1.14 (Danecek et al., 2011) with a 50 kb sliding window and a 25 kb sliding step across chromosomes. The top 5% *F*_ST_ values and *θ*_π_ ratios were considered as candidate selective sweeps regions.

### Candidate gene analysis

The candidate genomic regions were annotated using SNPeff v.5.1 (Cingolani et al., 2012) based on the goat reference genome (ARS1). Gene Ontology (GO) term enrichment and Kyoto Encyclopedia of Genes and Genomes (KEGG) pathway analyses of a candidate gene set were carried out by a statistical overrepresentation test with the default setting using the clusterProfiler 4.0 package in the R program (Wu et al., 2021). Categories with the threshold of adjusted *p*-value <0.05 after the Bonferroni correction were defined as significantly enriched terms and pathways.

## Results and discussion

**FIGURE 1:**
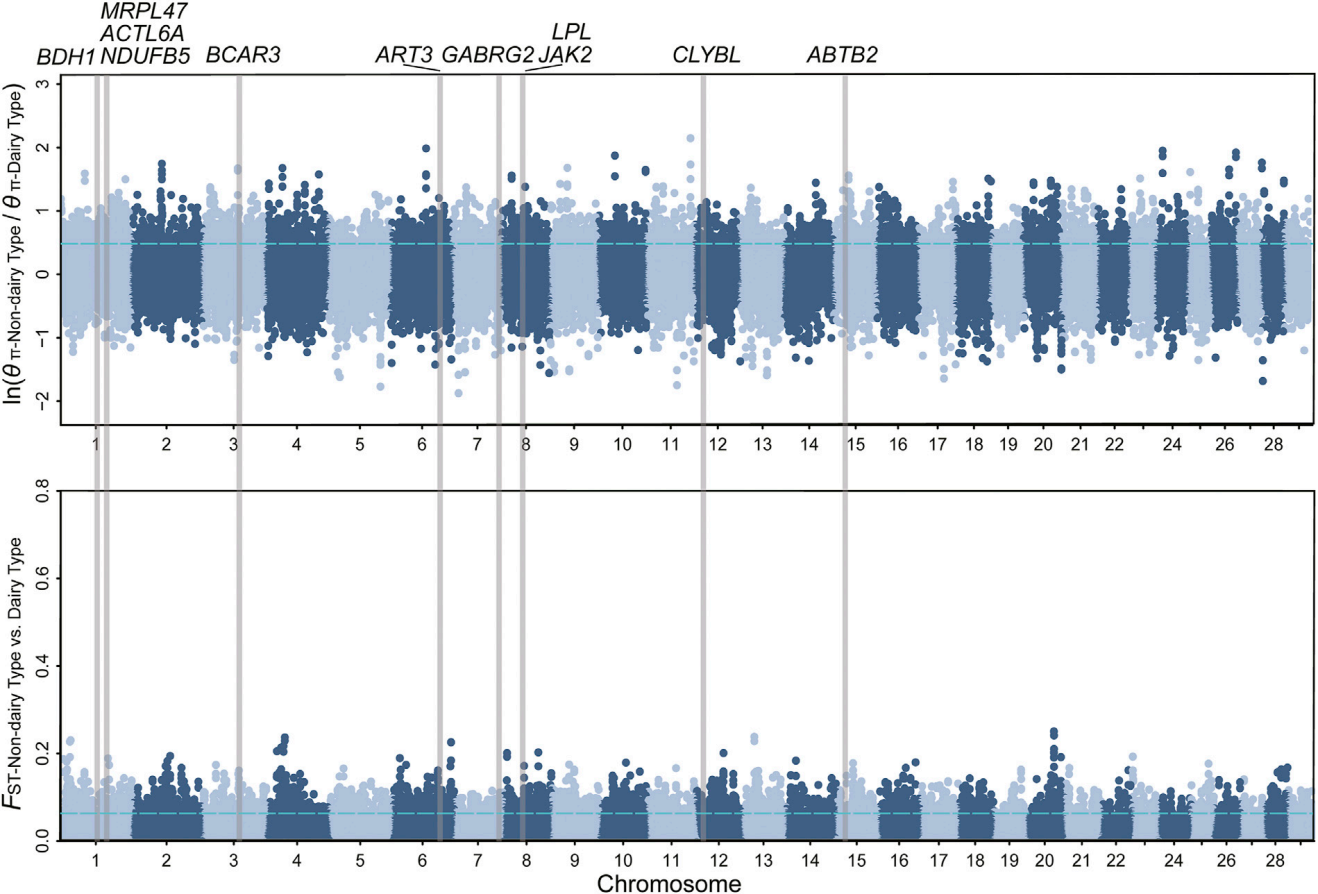
Manhattan plot of *θ*_π_ ratios and *F*_ST_ for milk traits. The vertical gray thick lines indicated the position of selected genes detected in our study. The values of the top 5% threshold (*F*_ST_ = 0.063, *θ*_π_ ratio = 0.488) are denoted by blue horizontal dashed lines.

**FIGURE 2:**
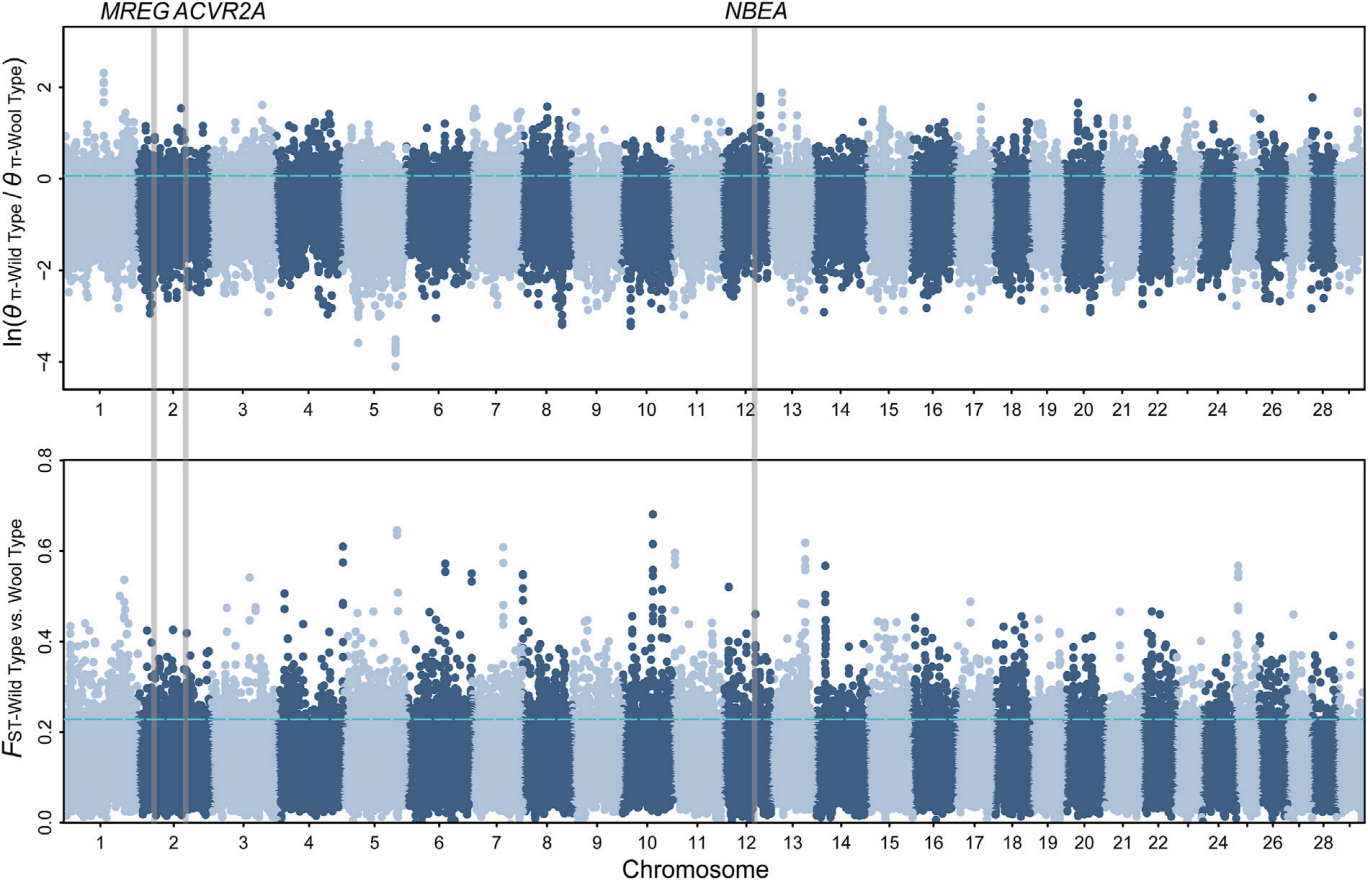
Manhattan plot of *θ*_π_ ratio and *F*_ST_ for wool traits. The vertical gray thick lines indicated the position of selected genes detected in our study. The top 5% threshold (*F*_ST_ = 0.228, *θ*_π_ ratio = 0.064) values are denoted by blue horizontal dashed lines.

**FIGURE 3:**
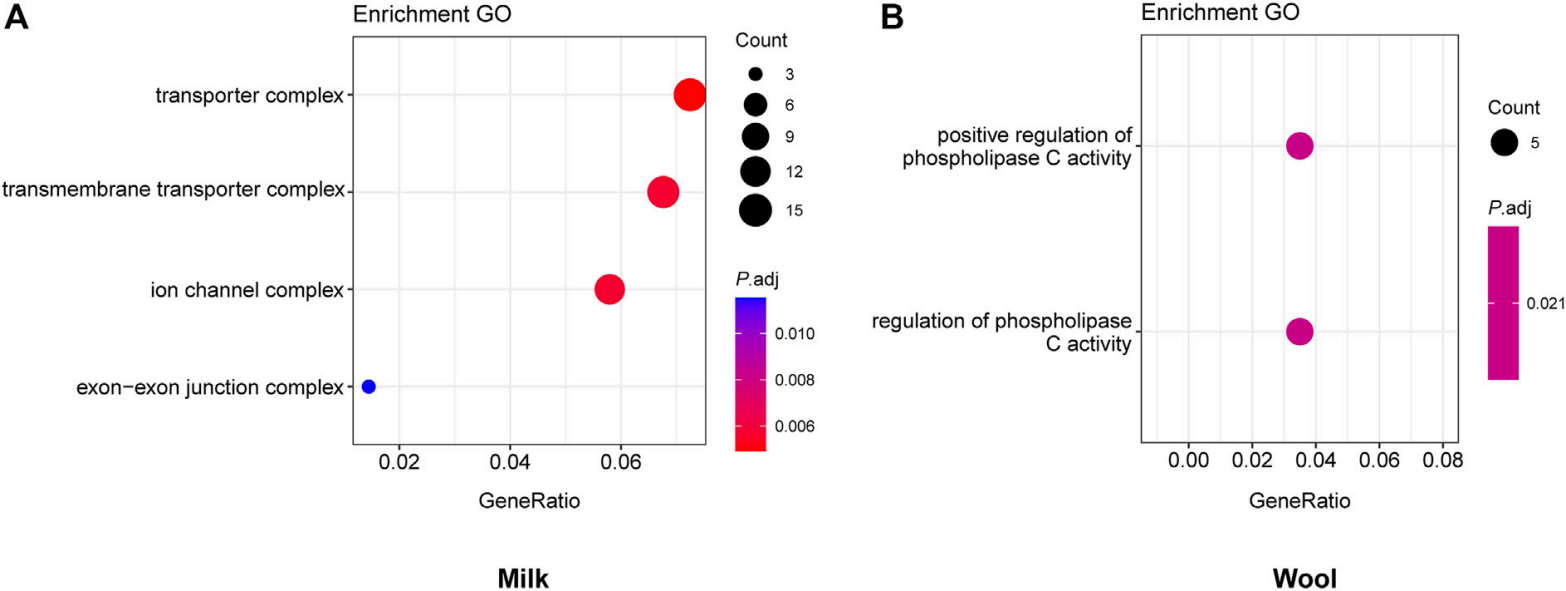
GO enrichment analyses for milk **(A)** and wool **(B)**, with the significant (*p*.adj <0.05) GO terms.

We obtained 28.56 million SNPs after filtering in 361 samples. We implemented selection screening in 6 pairs of populations (Table 1). 515, 303, 210, 531, 261, and 217 genomic regions were detected based on the overlap of the top 5% *θ*_π_ratio and *F*_ST_ for milk, wool, reproduction, horn, ear, and coat color traits, respectively. We further annotated those genomic regions and detected a set of novel and previously reported functional genes (Figures 1, 2; Supplementary Figure S1-S4; Supplementary Table S2-S7). 332 genes within 307 candidate selective sweep regions were identified to be associated with dairy traits (Supplementary Table S2). *LPL* was found to be associated with milk fat content in goats (Badaoui et al., 2007). Additionally, *BCAR3*, *ART3*, *GABRG2*, *JAK2,* and *ABTB2* were functionally associated with milk traits in cows (Gao et al., 2017; Khan et al., 2019; Zhou et al., 2019; Peters et al., 2021; Buaban et al., 2022). Similarly, *MRPL47*, *ACTL6A*, *NDUFB5,* and *BDH1* were identified as being related to milk yield in buffalo (El-Halawany et al., 2017; Du et al., 2019). We further investigated the functions of those genes by GO and KEGG analyses and found a significant overrepresentation of genes involved in GO terms, such as transporter complex, transmembrane transporter complex, and ion channel complex (Figure 3A). The GO term “ion channel” involved in the modulation of milk production by controlling mammary gland fluid flow in dairy cows (Cai et al., 2018). In the wool type *versus* wild type pair population, 203 genes within 188 candidate genomic regions were annotated and may be involved in the wool trait regulation mechanism (Supplementary Table S3). One of the strongest selective sweep signatures was situated in (neurobachin) *NBEA*, which encodes a neuron-specific multidomain protein of 327 kDa (Wang et al., 2000). Although the gene was a novel gene for goats, it had been reported to be associated with wool production in Chinese merino sheep (Wang et al., 2014). We detected a significant overrepresentation of genes in three GO and one KEGG terms involved Phospholipase C (PLC) activity (Figure 3B), and the reduction of PLC activity led to downregulation of keratin expression in mice hair, resulting in hair hypotrichosis and diverse hair anomalies (Nakamura et al., 2003; Nakamura et al., 2008). For the high prolificacy *versus* low prolificacy pair population, we obtained 164 functional genes within 142 genomic regions (Supplementary Table S4). *RELL1*, *KIT,* and *KITLG* were identified as candidate genes associated with prolificacy (Supplementary Figure S2). *RELL1* was reported to be associated with the number of stillborn in pigs (Onteru et al., 2012), the single nucleotide polymorphisms (SNPs) in *KIT* and *KITLG* were found to be associated with the litter size of goats and sheep (An et al., 2012; Yuan et al., 2019; Wang et al., 2020a). Further analysis showed the significant overrepresentation of genes in 12 GO terms, which are mainly associated with Second-messenger-mediated signaling and the activity of phospholipase (Supplementary Table S5A). The cyclic AMP governs the synthesis and secretion of reproductive hormones, particularly gonadotropins (Mukherjee and Mayo, 2000), and mediates the impacts of follicle-stimulating hormone and luteinizing hormone on the development of ovarian follicles and ovulation (Riccetti et al., 2018). 300 candidate genes were identified to be associated with poll traits (Supplementary Table S5). The highest peak of *θ*_π_ratio and *F*_ST_ is situated in *STIM1* and *NRXN1,* with *STIM1* involved in store-operated Ca^2+^ channels (Roos et al., 2005) and *NRXN1* binding neuroligins to form (Ca^2+^)-dependent neurexin/neuroligin complexes (Siddiqui et al., 2010) (Supplementary Figure S2). They both likely play important roles in neurodevelopment and behavior (Ching et al., 2010; Zheng et al., 2020). Further, GO enrichment analysis suggested a significant overrepresentation of genes involved in enamel mineralization (Supplementary Figure S5B). Enamel mineralization is involved in the process of producing and fortifying tooth enamel, the outermost layer of the tooth, which serves as a protective barrier (Vaissier Welborn, 2020), which may also have a correlation with the development and formation of the horn. In the big ear *versus* small ear goats, 205 genes within 188 genomic regions were identified to be associated with the big ear trait (Supplementary Table S6). In addition, we found a novel gene with big ear morphology in the goat. Furthermore, we detected a significant overrepresentation of genes involved in G protein−coupled purinergic nucleotide receptor activity, purinergic nucleotide receptor activity, and nucleotide receptor activity (Supplementary Figure S5C), potentially exerting an influence on ear morphology. Overall, 145 genes within 146 genomic regions were identified in the white coat color *versus* black coat color pair population (Supplementary Table S7). The two genes *AHCY*, and *EDNRA* were identified to be involved in the regulation of coat color (Menzi et al., 2016; Nazari-Ghadikolaei et al., 2018) (Supplementary Figure S4). We also detected novel genes involved with coat colors in goats, such as *GPR22*, *SOX5*, *CLEC12B*, *AIM1*, *ITFG1*, and *LDLRAD4* (Table 2).

**TABLE 2:** Candidate genes under selection based on pairwise *F*_ST_ and *θ*_πratio_.

Traits	Comparisons	Candidate genes
Milk	Dairy type *versus* Non-dairy type	*BDH1, MRPL47, ACTL6A, NDUFB5, BCAR3, ART3, GABRG2, CLYBL, ABTB2*
Wool	Wool type *versus* Wild type	*MREG, ACVR2A, DKK2, NBEA*
Reproduction	High prolificacy *versus* Low prolificacy	*PDIA4, KITLG, RELL1, KIT, NFIC, DMRT1, BIRC6, SPIRE2*
Horn	Poll *versus* Horn	*STIM1, NRXN1*
Ear	Big ear *versus* Small ear	*LEP*
Coat Color	White *versus* Black	*GPR22, SOX5, CLEC12B, AIM1, AHCY, EDNRA, ITFG1, LDLRAD4*

The italic in column “candidate genes” indicate name of gene.

In summary, we collected diverse goats’ germplasm resources and explored functional genes of important phenotypical traits. Our study confirmed previous results and also identified some novel genes involved in the regulation of specific traits. Our study provides deep insights into the genetic mechanisms of complex traits and genetic markers for genetic improvement in goats.

## Data Availability

The datasets presented in this study can be found in online repositories. The names of the repository/repositories and accession number(s) can be found in the article/Supplementary Material.
