# Supplementary material for: Whole-genome selective scans detect genes associated with important phenotypic traits in goat (Capra hircus)
Source: Front Genet. 2023 Apr 18;14:1173017. doi: 10.3389/fgene.2023.1173017 (PMC10151485; doi:10.3389/fgene.2023.1173017)
Supplement: Supplementary file 2 [file Image1.pdf]

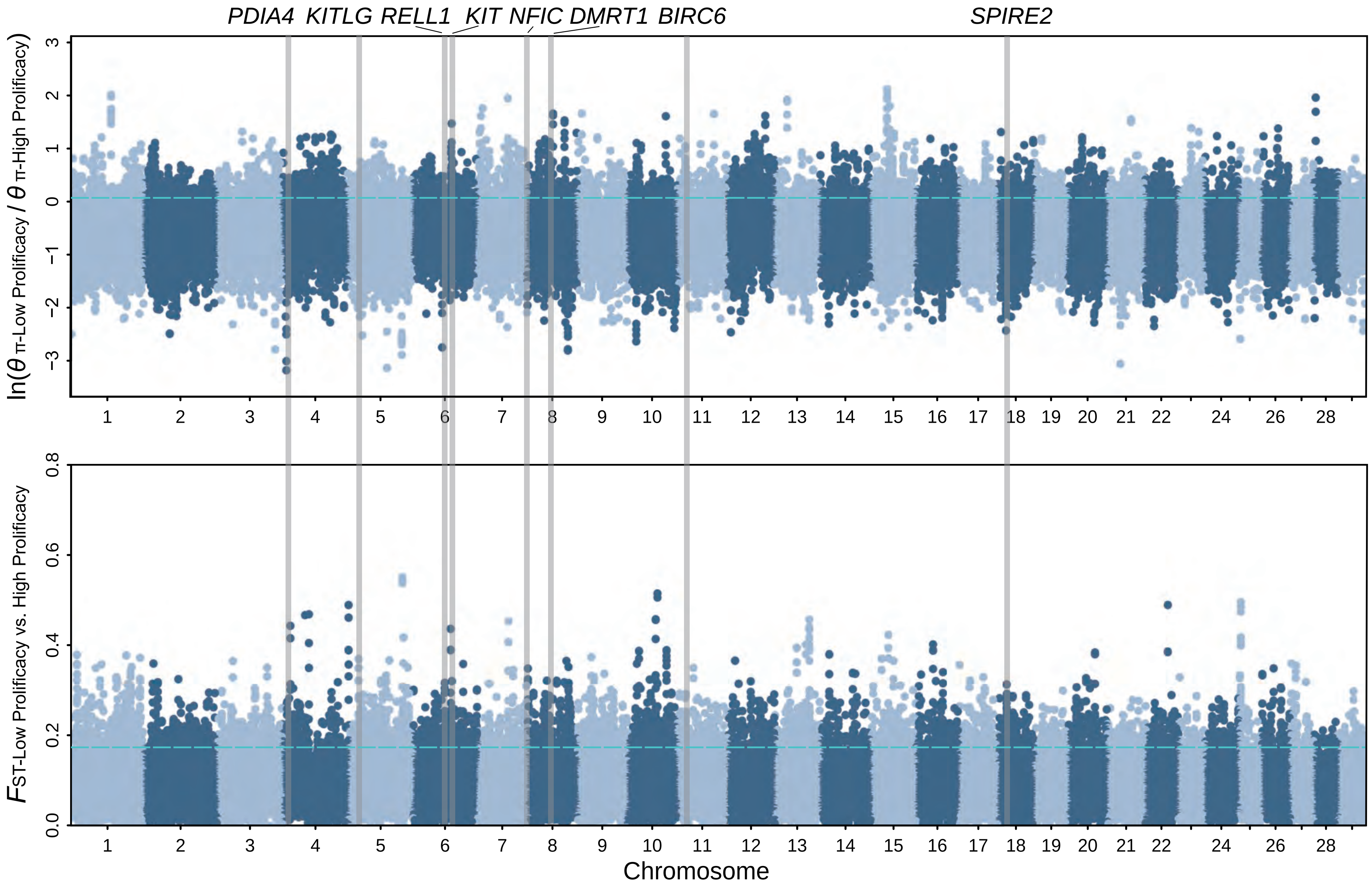


**Figure S1** Manhattan plot of $\theta_\pi$ ratio and $F_{ST}$ for prolificacy traits. The vertical gray thick lines indicated the position of selected genes detected in our study. The top 5% threshold ($F_{ST}$ = 0.173, $\theta_\pi$ ratio = 0.064) values are denoted by blue horizontal dashed lines.

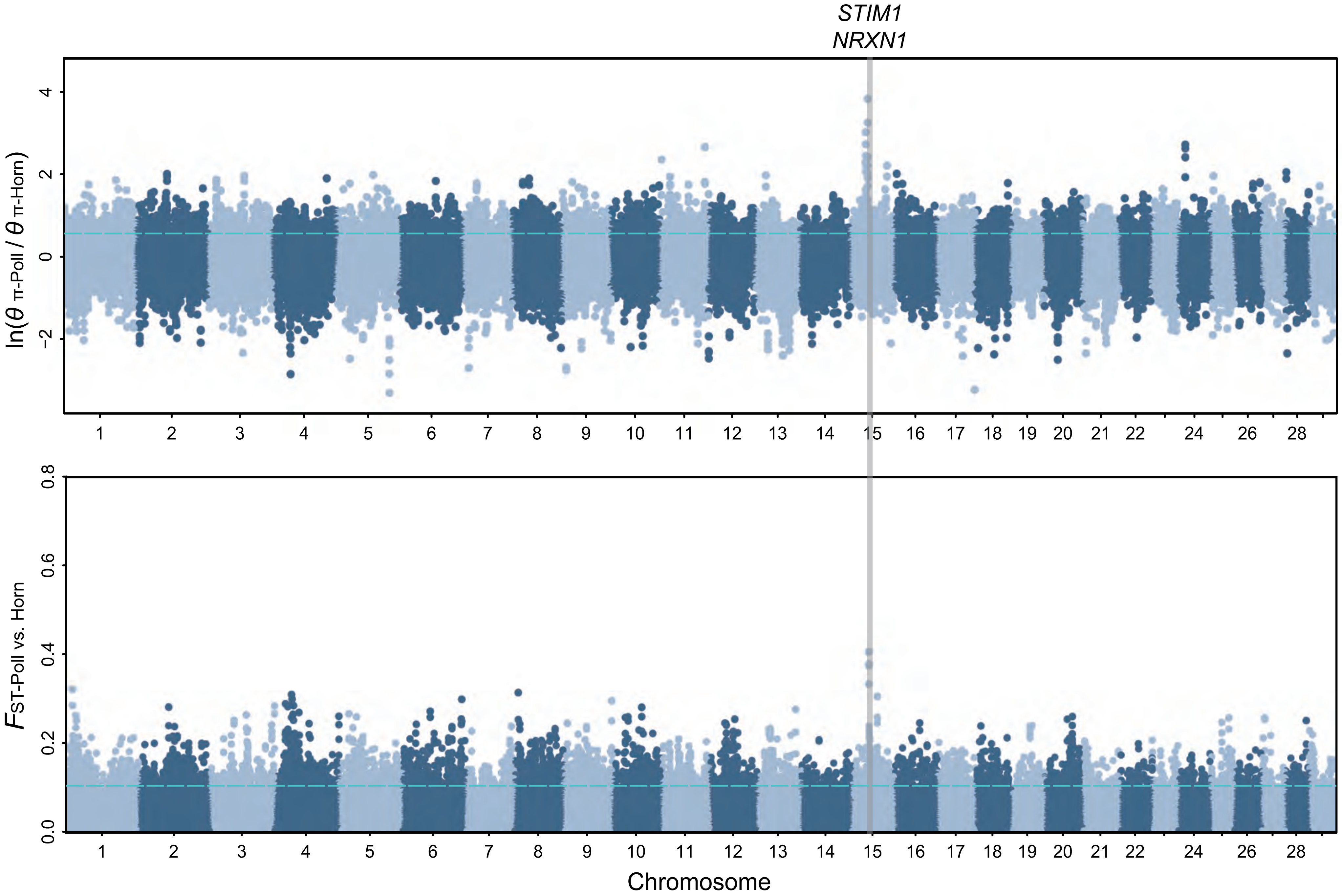


**Figure S2** Manhattan plot of $\theta_\pi$ ratio and $F_{ST}$ for horn traits. The vertical gray thick lines indicated the position of selected genes detected in our study. The top 5% threshold ($F_{ST}$ = 0.107, $\theta_\pi$ ratio = 0.563) values are denoted by blue horizontal dashed lines.

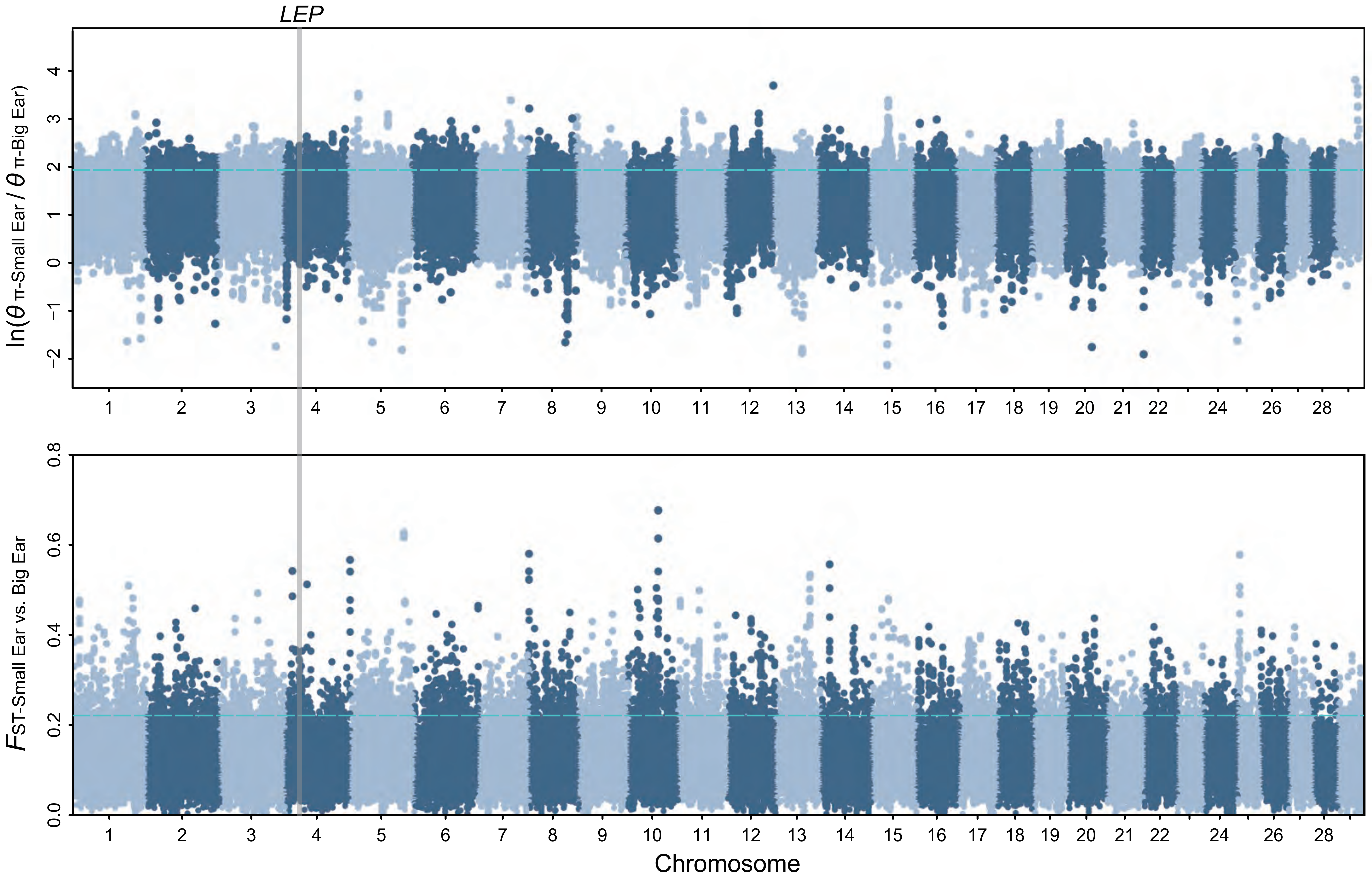


**Figure S3** Manhattan plot of $\theta_\pi$ ratio and $F_{ST}$ for ear traits. The vertical gray thick lines indicated the position of selected genes detected in our study. The top 5% threshold ($F_{ST}$ = 0.220, $\theta_\pi$ ratio = 1.932) values are denoted by blue horizontal dashed lines.

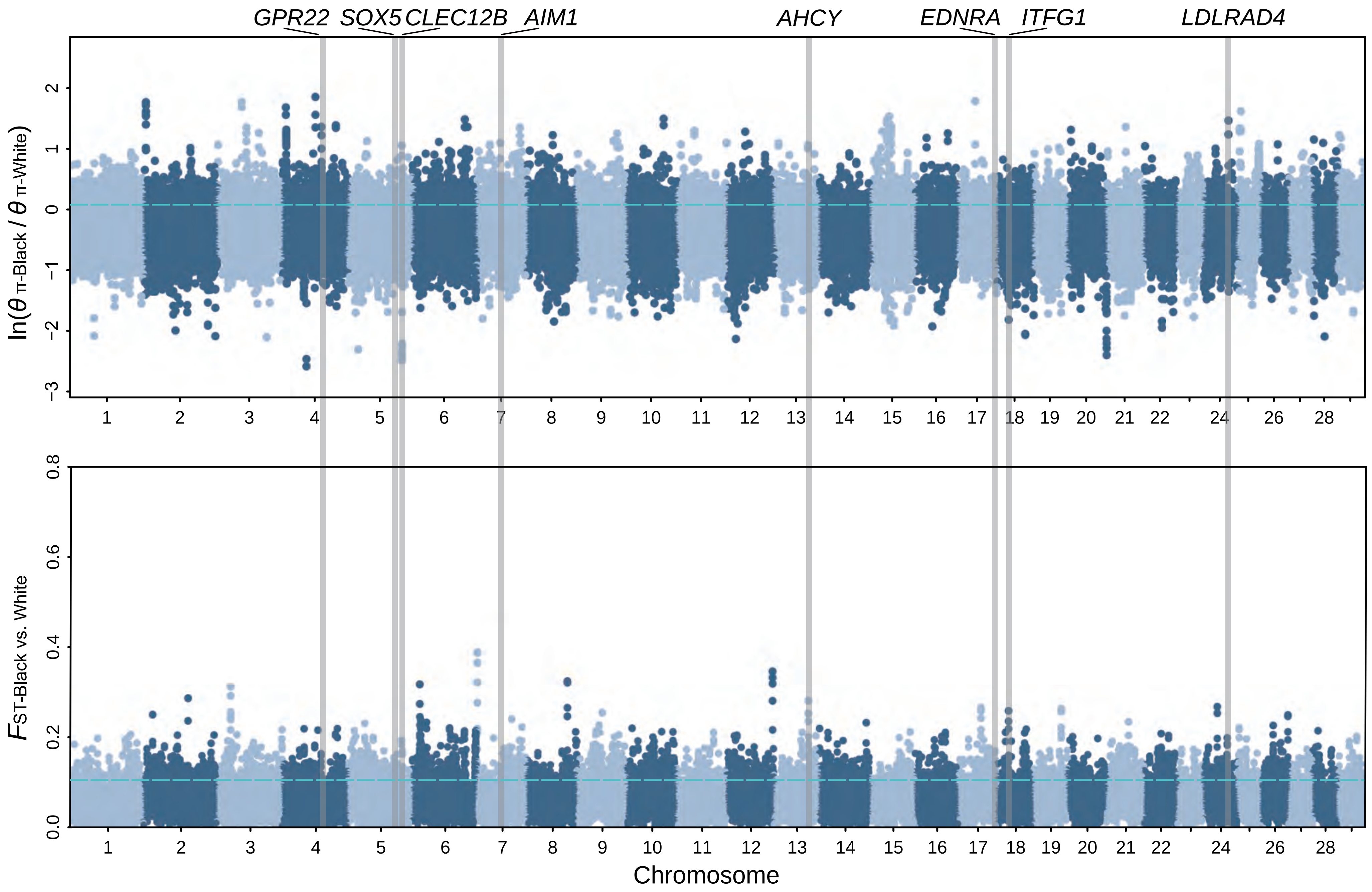


**Figure S4** Manhattan plot of $\theta_\pi$ ratio and $F_{ST}$ for coat color traits. The vertical gray thick lines indicated the position of selected genes detected in our study. The top 5% threshold ($F_{ST}$ = 0.104, $\theta_\pi$ ratio = 0.159) values are denoted by blue horizontal dashed lines.

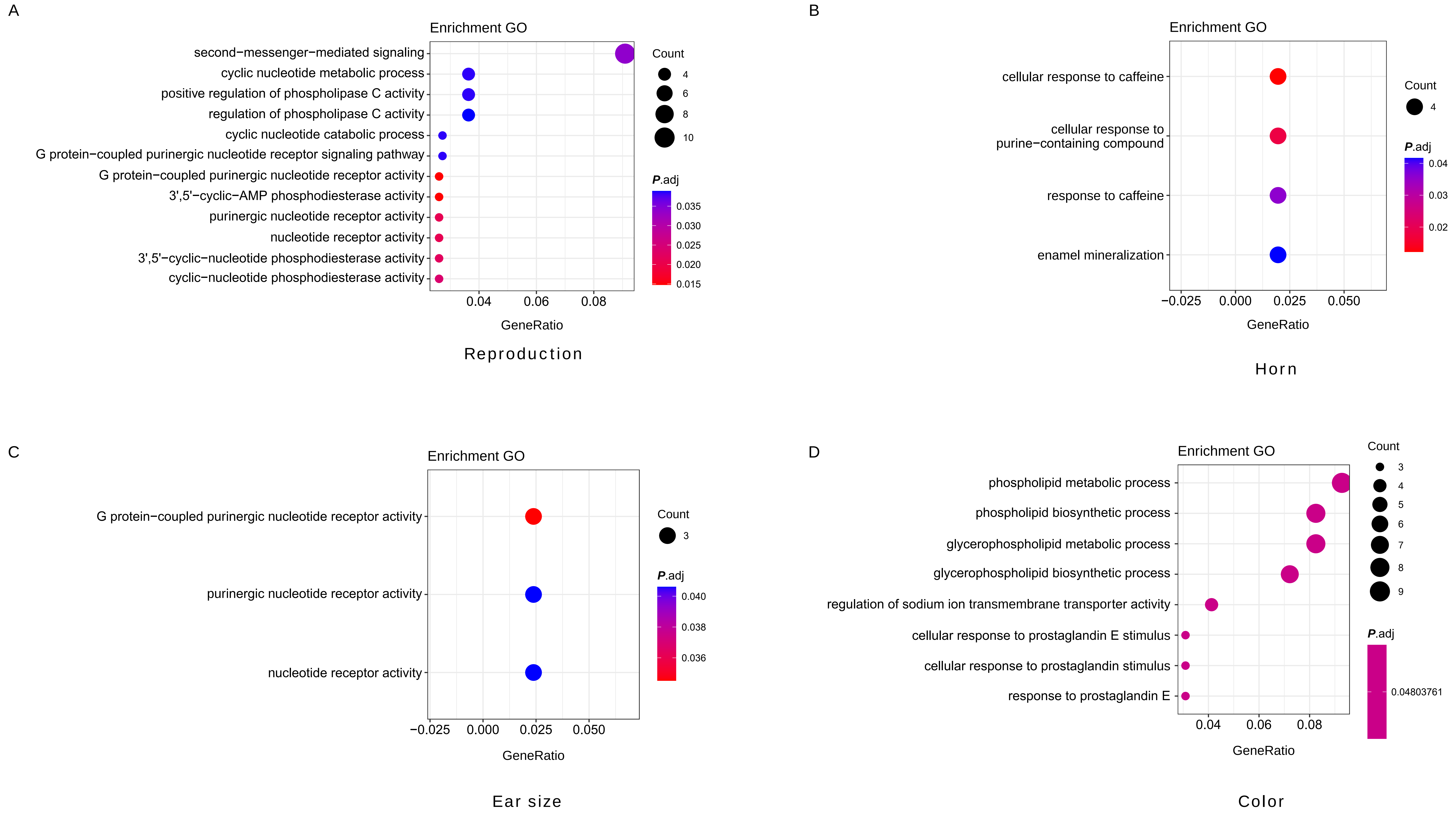


**Figure S5** GO enrichment analyses for high prolificacy (**A**), poll (**B**), big ear size (**C**), and white coat color (**D**), with the significant ($P_{adj} < 0.05$) GO terms.
